# Supplementary material for: A Facile Synthesis of Noble-Metal-Free Catalyst Based on Nitrogen Doped Graphene Oxide for Oxygen Reduction Reaction
Source: Materials (Basel). 2022 Jan 21;15(3):821. doi: 10.3390/ma15030821 (PMC8837119; doi:10.3390/ma15030821)
Supplement: Supplementary file 1 [file materials-15-00821-s001.zip › materials-1532657-supplementary.pdf]

Supplementary Materials

A Facile Synthesis of Noble-Metal-free Catalyst Based on Nitrogen Doped Graphene Oxide for Oxygen Reduction Reaction

Vladimir P. Vasiliev [1,*], Roman A. Manzhos [1], Valeriy K. Kochergin [1], Alexander G. Krivenko [1], Eugene N. Kabachkov [1,2], Alexander V. Kulikov [1], Yury M. Shulga [1] and Gennady L. Gutsev [3,*]

[1] Institute of Problems of Chemical Physics of RAS, Acad. Semenov ave., 1, Chernogolovka 142432, Russia; rmanzhos@yandex.ru (R.A.M.); kocherginvk@yandex.ru (V.K.K.); krivenko@icp.ac.ru (A.G.K.); en.kabachkov@gmail.com (E.N.K.); kulav@icp.ac.ru (A.V.K.); yshulga@gmail.com (Y.M.S.)
[2] Chernogolovka Scientific Center, Russian Academy of Sciences, Chernogolovka 142432, Russia
[3] Department of Physics, Florida A&M University, Tallahassee, FL 32307, USA
* Correspondence: vpvasiliev@mail.ru (V.P.V.); gennady.gutsev@famu.edu (G.L.G.)

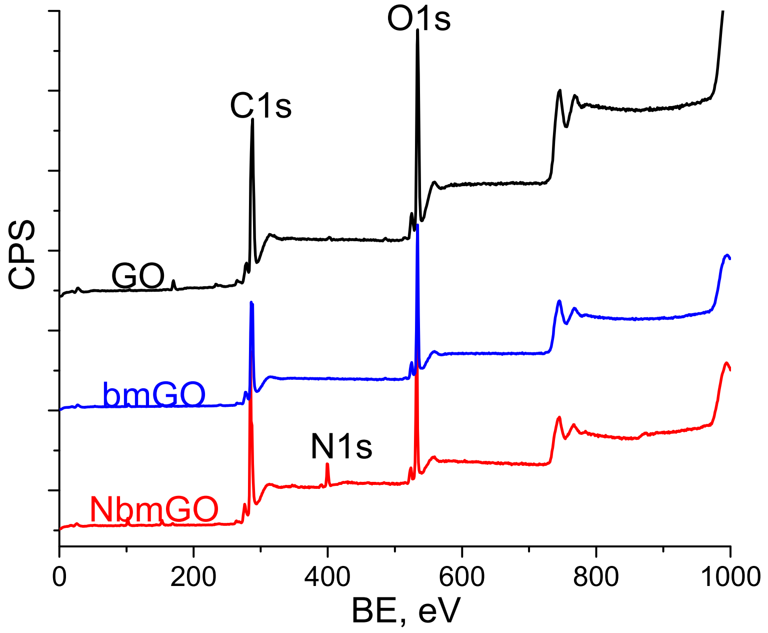

Figure S1. XPS survey spectra of the GO, bmGO, and NbmGO samples.

Table S1. Elemental composition (in at.%) of the samples under study.

Samples	Elements, %			
	C	N	O	S
GO	74.3	0.3	23.5	1.9
bmGO	76.8	0.0	23.0	0.2
NbmGO	76.7	5.5	17.4	0.4

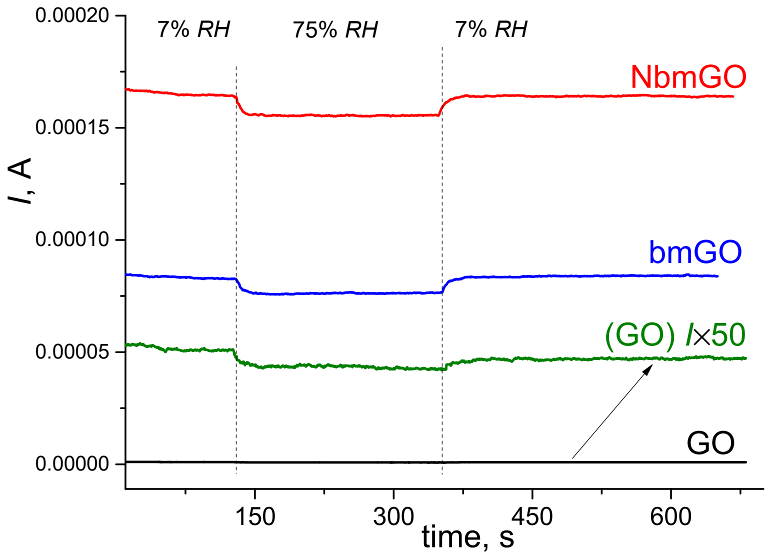

Figure S2. The conductivity of GO, bmGO, and NbmGO films at different *RH*%.

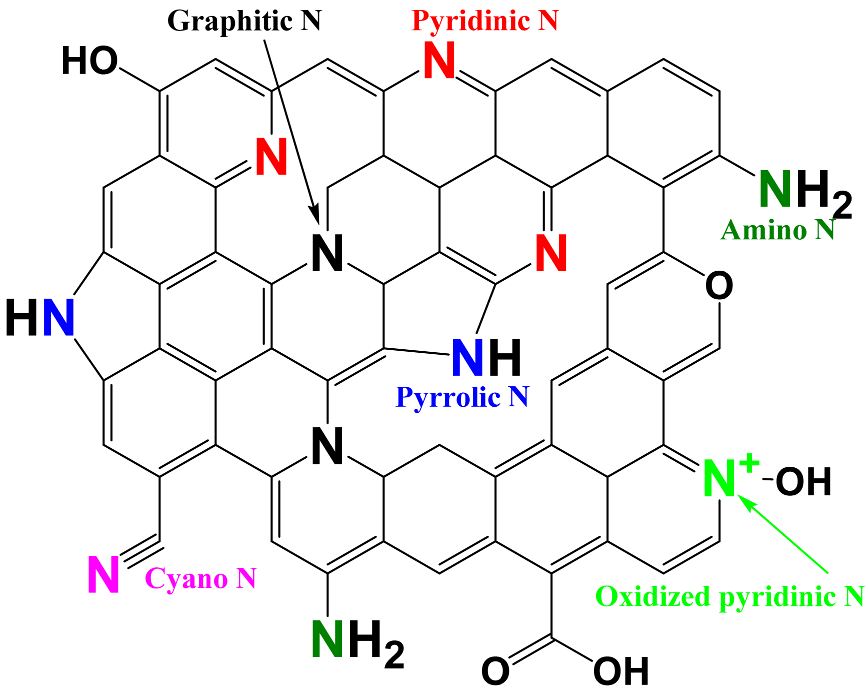

Figure S3. A model structure of NbmGO.

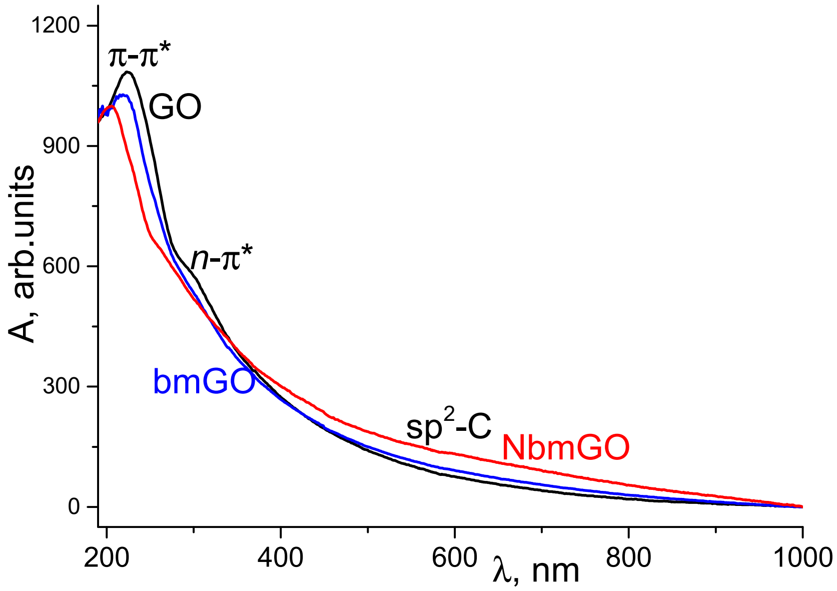

Figure S4. Electronic absorption spectra of GO, bmGO and NbmGO films (quartz glass). The maximum of the electronic absorption spectrum shifts to shorter wavelengths (224 nm → 205 nm) for the samples of GO, bmGO and NbmGO and there is an increase in absorption observed in the range 400-800 nm, which is typical for sp^2-carbon.

Table S2. Positions (Eb), full widths at half maximum (FWHM), and intensities (Int) of the peaks in the XPS spectra of the GO, bmGO and NbmGO samples

Samples	Line (Peak)	E_b, eV	*FWHM*, eV	*Int*, %	*Assignment*
GO	C1*s*(C1)	284.6	1.70	40.7	C=C/C–C
	C1*s*(C2)	286.6	1.65	41.6	C–O/C–OH
	C1*s*(C3)	288.1	2.00	15.2	C=O
	C1*s*(C4)	289.7	1.90	2.5	O–C=O
	O1*s*	533.5	2.10	100	
bmGO	C1*s*(C1)	284.6	1.45	46.2	C=C/C–C
	C1*s*(C2)	286.7	1.40	43.1	C–O/C–OH
	C1*s*(C3)	288.4	1.80	9.5	C=O
	C1*s*(C4)	290.1	1.65	1.2	O–C=O
	O1*s*	533.8	2.00	100	
NbmGO	C1*s*(C1)	284.7	1.40	57.1	C=C/C–C
	C1*s*(C2)	286.7	1.65	31.1	C–O/C–OH
	C1*s*(C3)	288.6	1.95	10.2	C=O
	C1*s*(C4)	290.6	1.90	1.6	O–C=O
	O1*s*	533.9	2.00	100	
	N1*s*(N1)	398.5	1.50	19.4	Pyridinic N
	N1*s*(N2)	399.6	2.00	67.6	Pyrrolic N
	N1*s*(N3)	401.0	2.60	13.0	Graphitic

Table S3. Peak positions and the band intensity ratios (ID/IG) in the Raman spectra of graphite, GO, bmGO, and NbmGO.

Samples	Peak position, cm^{-1}		I_D/I_G
	D	*G*	
**Graphite [1]**	1350	1583	–
GO	1350	1599	0.864
bmGO	1348	1598	0.962
NbmGO	1344	1595	1.011

Table S4. ESR data obtained for samples of GO, bmGO, and NbmGO at room temperature.

Samples	g-factor	Line widthsΔH, mT (G)	Form-factor FF	The number of paramagnetic centers*N*, spin/g
GO	2.0048	0.17 (1.7 G)	3.4	5.4×10^{17}
bmGO	2.0027	0.25 (2.5 G)	3.4	5.5×10^{18}
NbmGO	2.0034	0.39 (3.92 G)	3.6	6.2×10^{18}

Table S5. The characteristic features in the FTIR spectra of GO and melamine

Absorption bands (cm^{-1})	Description
	**GO** (see Ref. S2–S8)
3000–3700	stretching vibrations of O–H
1720–1730	stretching vibrations of C=O carbonyl groups and/or ketones
1620–1630	vibrations of double C=C bonds and bending vibrations of the water molecules
1220–1230	C–OH stretching
1040–1060	vibrations epoxy and alkoxy groups
	**Melamine** (see Ref. S9–S10)
3468	stretching vibrations of N–H
3417	stretching vibrations of N–H
3324	stretching vibrations of N–H
3121	stretching vibrations of N–H
1630–1660	NH_2 deformation

Table S6. Electrochemical properties of N-doped carbon materials.

No	Catalyst	Half-wave potential/Onset potential (V vs. Ag/AgCl)	Ref. SI
1	NbmGO	–0.175/–0.09	This work
2	bmGO	–0.215/–0.105	This work
3	rGOA-MS	–0.295/–0.15	[11]
4	NG5	–0.25/–0.1	[12]
5	NG-C	–0.2/–0.06	[13]
6	PDI-900	–0.215/–0.14	[14]
7	NrGO800	–0.205/–0.08	[15]

The chronoamperometry test of NbmGO catalyst was carried out at 2000 rpm in O_2-saturated 0.1 M KOH solution (Fig. SI5). The high current retention of 93 % after continuous polarization at –250 mV during 720 min clearly demonstrates the excellent long-term stability of NbmGO, which can be attributed to the quite little loss of active sites during the test.

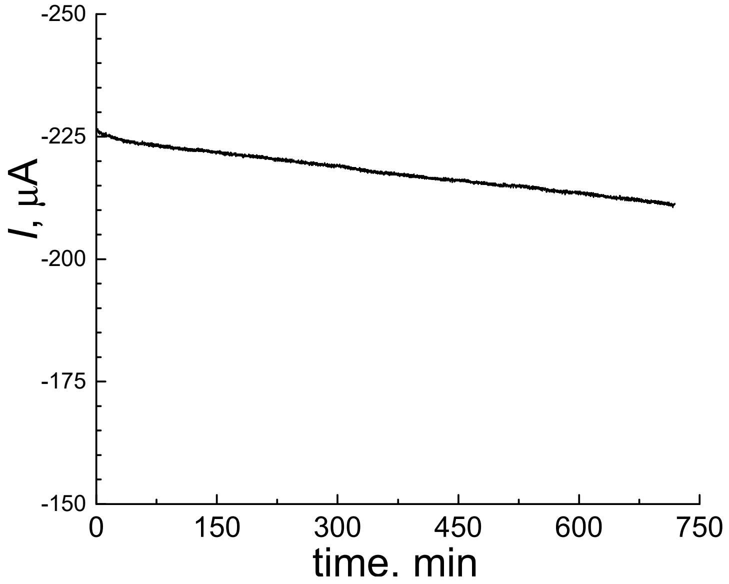

Figure S5. Long-term stability of NbmGO *via* chronoamperometry test at –300 mV in 0.1 M KOH and at room temperature, ω = 2000 rpm.

Materials and Synthesis of GO

All the materials were used without further purification. Graphite powder (<50 μm, Merck (Germany), 98+%), potassium permanganate ($KMnO_4$, Rushim (Russia), 98%), concentrated sulfuric acid (H_2SO_4, Rushim (Russia), 99%), nitric acid (HNO_3,Rushim (Russia), 99%), 30% hydrogen peroxide (H_2O_2, Chimmed (Russia), 98%), 37% hydrochloric acid (HCl, Rushim (Russia), 99%). Melamine ($C_3N_6H_6$, BASFSE (Germany), 99.9%).Graphite oxide was prepared by Hammers [16], with some modification [17].The elemental composition of the product corresponded to a formula $C_8O_{4.6}H_{1.8}(H_2O)_{0.58}$. According to X-ray diffraction study, the interplanar distance is 0.81 nm in our sample.

References S:

[S1] Odake, S.; Zinin, P.V.; Hellebrand, E.; Prakapenka, V.; Liu, Y.; Hong, S.; Burgessa, K.; Ming, L-C. Formation of the high-pressure graphite and BC_8 phases in a cold compression experiment by Raman scattering, *J. Raman Spectrosc*., **2013**, 44, 1596–1602.

[S2] Si, Y.; Samulski, E.T. Synthesis of water soluble graphene, *Nano Lett*., **2008**, 8, 1679–1682.

[S3] Jeong, H.K.; Lee, Y.P.; Jin, M.H.; Kim, E.S.; Bae, J.J.; Lee, Y.H. Thermal stability of graphite oxide, *Chem. Phys. Lett*., **2009**, 470, 255–258.

[S4] Cote, L.J.; Cruz-Silva, R.; Huang, J. Flash reduction and patterning of graphite oxide and its polymer composite, *J. Am. Chem. Soc*., **2009**, 131, 11027–11032.

[S5] Karthika, P.; Rajalakshm,i N.; Dhathathreyan, K.S. Functionalized exfoliated graphene oxide as supercapacitor electrodes, *Soft Nanosci. Lett*., **2012**, 2, 59–66.

[S6] Galande, C.; Mohite, A.D.; Naumov, A.V.; Gao, W.; Ci, L.; Ajayan, A.; Gao, H.; Srivastava, A.; Weisman, R.B.; Ajayan, P.M. Quasi-molecular fluorescence from graphene oxide, *Scientific Reports*, **2011**, vol. 1, p. 85.

[S7] Fu, M.; Jiao, Q.; Zhao, Y.; Li, H.; Vapor diffusion synthesis of $CoFe_2O_4$ hollow sphere/graphene composites as absorbing materials, *J. Mater Chem. A*, **2014**, 2, 735–744.

[8] Smith, A.L. Applied Infrared Spectroscopy. John Wiley and Sons, Ltd., Chichester, Sussex, **1979**, 322 p.

[S9] Wang, Y-L.; Mebel, A.M.; Wu, C-J.; Chen, Y-T.; Lina, C-E.; Jiang, J-C. IR spectroscopy and theoretical vibrational calculation of the melamine molecule J. Chem. Soc., *Faraday Trans*., **1997**, 93, 3445–3451.

[S10] Costa, L.; Camino, G. Thermal behaviour of melamine, *Journal of Thermal Analysis*, **1988**, 34, 423–429.

[S11] Manzhos, R.A.; Baskakov, S.A.; Kabachkov, E.N.; Korepanov, V.I.; Dremova, N.N.; Baskakova, Y.V.; Krivenko, A.G.; Shulga, Y.M.; Gutsev G.L. Reduced graphene oxide aerogel inside melamine sponge as an electrocatalyst for the oxygen reduction reaction, *Materials*, **2021**, *14*, 322, 12 p.

[S12] Sheng, Z-H.; Shao, L.; Chen, J-J.; Bao, W-J.; Wang, F-B.; Xia,X-H. Catalyst-Free Synthesis of Nitrogen-Doped Graphene *via* Thermal annealing graphite oxide with melamine and its excellent electrocatalysis, *A*CS Nano, **2011**, vol. 5, p. 4350–4358.

[S13] Liao, Y.; Gao, Y.; Zhu, S.; Zheng, J.; Chen,Z.; Yin,C.; Lou,X.; Zhang,D. Facile fabrication of N-doped graphene as efficient electrocatalyst for oxygen reduction reaction, *ACS Appl. Mater. Interfaces*, **2015**, 7, 35, 19619–19625.

[S14] Liu, R.; Wu, D.; Feng, X.; Müllen, K.; Nitrogen-doped ordered mesoporous graphitic arrays with high electrocatalytic activity for oxygen reduction, *Angew. Chem. Int. Ed*., **2010**, 49, 2565–2569.

[S15] Lemesa, G.; Sebastiana, D.; Pastorb, E.; Lazaro, M.J. N-doped graphene catalysts with high nitrogen concentration for the oxygen reduction reaction, Journal of Power Sources, 2019, 438, 227036, 10 p.

[S16] Hummers, W.S.; Offeman, R.E. Preparation of graphitic oxide, *J. Am. Chem. Soc*., **1958**, 80, 1339.

[S17] Muradyan, V.E.; Ezernitskaya, M.G.; Smirnova, V.I.; Kabaeva, N.M.; Novikov, Y.N.; Parnes, Z.N.; Volpin, M.E. Transformation of Graphitic oxide under ionic hydrogenation conditions, *Russ. J. Gen. Chem*., **1991**, 61, 2433–2435.
